# The relationship between alcohol consumption, social distancing, and crime rates: insights from the COVID-19 pandemic

**DOI:** 10.7189/jogh.16.04144

**Published:** 2026-04-30

**Authors:** Agnus M Kim, Jin-Seok Lee, Doojin Ryu

**Affiliations:** 1 Department of Preventive Medicine, Hanyang University College of Medicine, Seoul, Republic of Korea; 2 Department of Health Policy and Management, Seoul National University College of Medicine, Seoul, Republic of Korea; 3 Medical Research Center, Institute of Health Policy and Management, Seoul National University, Seoul, Republic of Korea; 4 Department of Economics, Sungkyunkwan University, Seoul, Republic of Korea

## Abstract

**Background:**

While there was a global shift in social interaction and alcohol consumption during the COVID-19 pandemic, their associations with changes in crime rates remain underexplored. We aimed to examine the associations between crime rates and alcohol use within the context of pandemic-related social distancing.

**Methods:**

We calculated crime rates across crime categories from 2011 to 2022 using crime statistics from the Korean National Police Agency. We estimated two linear regression models with the crime rate as the dependent variable. The first model examined the association of movie attendance (a proxy for social distancing) and the unemployment rate with crime rates. The second model additionally included *per capita* alcohol consumption to determine how the association between social distancing and crime rates was attenuated when accounting for alcohol use.

**Results:**

As of 2022, 19% of total crimes involved offenders under the influence of alcohol, with particularly high proportions in murder (64%), traffic accidents (47%), arson (32%), violence (28%), and rape (20%). Overall crime rates and offences committed under the influence of alcohol, which had steadily declined from 2011, fell sharply during the COVID-19 pandemic. Both alcohol consumption and social interaction declined during the pandemic. While the rates of overall crime, violence, rape, traffic accidents, and arson were initially associated with social contact, these associations were no longer significant after adjusting for alcohol consumption; instead, strong positive associations with alcohol consumption were observed. The rate of murder was not significantly associated with social contact, but exhibited a significant association only with alcohol consumption.

**Conclusions:**

The concurrent declines in crime and alcohol consumption, along with the attenuating effect of alcohol in the relationship between social distancing and crime, suggest that addressing social drinking environments may be an effective strategy for reducing crime rates.

Alcohol is widely recognised as a contributing factor to criminal behaviour. It impairs judgement and self-control [1], thereby increasing the risk of aggressive behaviour [2]. A substantial proportion of crimes, particularly violent offences, are committed under the influence of alcohol [3], with estimates indicating that one-third to half of violent crimes and sexual assaults in industrialised nations involve alcohol use [2,4].

The causal relationship between alcohol consumption and crime has been investigated from various perspectives. Natural experiments leveraging geographic and temporal variations in alcohol taxes, pricing, drinking age, and availability have demonstrated significant associations [5,6]. However, these studies frequently focused on limited populations, typically confined to specific regions, underscoring the need for broader investigations. In this context, the COVID-19 pandemic offered a unique opportunity to examine changes in alcohol consumption across the entire population.

There was a worldwide decrease in crime during the pandemic [7], except for domestic crimes [8–11], homicides [12–14], and cybercrimes [15–18], which showed mixed results depending on the region and the period of the pandemic. Reduced interpersonal contacts – whether from stay-at-home policies and other social restrictions [7,19] or voluntary behaviour changes [20] – may have disrupted the convergence of potential offenders, suitable targets, and the absence of guardians required for crime, as proposed by routine activity theory [7,21]. Considering that circumstantial factors conducive to crime are critical for its occurrence, as suggested by situational crime prevention or opportunity-based theories, measures such as mobility restrictions, limits on public gatherings, increased policing, and improved reporting systems may have unintentionally reduced opportunities for crime [22,23].

However, despite the persuasiveness of this argument, existing analyses of changes in crime during the pandemic in relation to social distancing have overlooked a significant contributor to crimes: alcohol. Given the substantial role of intoxication in the occurrence of crimes and the potentially strong impact of social distancing on alcohol consumption, changes in crimes during the pandemic need to be examined in relation to alcohol consumption.

The pathway by which alcohol influences the occurrence of crime can be explained from individual and contextual perspectives. Biologically, alcohol impairs judgement, cognition, and self-inhibition, and increases aggression, making individuals more likely to commit crimes. Therefore, a reduction in alcohol consumption may decrease the likelihood of crime. Alcohol consumption may also influence the occurrence of crime at the contextual level. The environments in which drinking occurs and the act of drinking itself can reduce individuals’ motivation to control their behaviour and provide an excuse for deviant behaviour. The places or atmospheres in which drinking occurs are also susceptible to violence, in addition to the increased likelihood of interpersonal friction that may arise during social gatherings. The impact of the pandemic on alcohol consumption was mixed. While global alcohol consumption overall declined, diverse findings were reported across countries [24,25]. These mixed findings may be attributed to differences in drinking culture, the mental health effects of the pandemic, and variations in alcohol regulations. However, a long-term downward trend in alcohol consumption was more commonly observed in most countries [25], and its effect on crime warrants examination. Among the countries affected, Korea presents a particularly compelling case due to both its high alcohol consumption and deeply rooted drinking culture.

In Korea, drinking plays a central role in formal and informal interactions, with binge drinking being especially widespread [26]. Although Korea ranks in the upper quartile worldwide for *per capita* alcohol intake [27], the social costs associated with alcohol use far exceed those of other developed countries with similar or higher alcohol consumption levels [28]. We aim to examine changes in crime rates and alcohol consumption during the pandemic in the context of social distancing, providing robust evidence of the role of alcohol in crime, particularly as it relates to social drinking behaviours.

## METHODS

### Variables and data

We obtained crime statistics from 2011 to 2022 from the Korean National Police Agency [29], based on nationwide reports from all police offices in Korea. The agency categorises crimes as murder (completed and attempted), traffic accidents, rape, arson, theft, violence, public order offences, and intellectual crimes (*i.e.* white-collar offences including fraud, embezzlement, and forgery). The agency further classifies crime statistics according to the perpetrator’s mental status, including normal, mental disorder (specified as schizophrenia), intellectual disability (categorised as mental retardation in the original data set), other mental disorders (defined as bipolar disorder or personality disorder), intoxication, and menstruation-related conditions [30]. The police officer assesses the mental status of the perpetrator based on the perpetrator’s condition at the time of the offence and, if necessary, medical records. The police officer also determines intoxication through a rigorous assessment of various circumstantial factors, including physiological signs of the suspect (*i.e.* facial flushing, alcohol smell, unsteady gait, and slurred speech), as well as statements from the suspect and witnesses. While intoxication in traffic accidents is measured via a breath test (blood alcohol concentration ≥0.03%), crime statistics only report the number of crimes committed under the influence across all crime types without specifying blood alcohol levels, and we calculated the rate of such crimes based on this classification.

To control for economic factors, we sourced the unemployment rate (for ages ≥15) from Statistics Korea [31]. We used the annual movie attendance data from the Korean Film Council as a proxy for social distancing measures [32]. Movie attendance dropped to approximately one-quarter of pre-pandemic levels during the first year of the COVID-19 pandemic and remained relatively stable the following year [32], making it a reliable indicator of social distancing compared to other proxy measures, such as overseas departures and domestic trips [33,34]. We estimated the *per capita* annual alcohol consumption (for ages ≥20) from National Tax Service alcohol shipment data, which comprised domestically manufactured products and imported alcoholic beverages. We adhered to the STROBE reporting guidelines for observational studies (Table S1 in the **Online Supplementary Document**) [35].

### Statistical analysis

We analysed crime rates per 100 000 population for murder (completed and attempted), traffic accidents, rape, arson, theft, violence, public order offences, and intellectual crimes from 2011 to 2022. Because data before 2013 combined rape, attempted rape, and sexual harassment, we calculated rape rates starting from 2013. We assessed overall crime rates and crimes committed under the influence of alcohol. We calculated correlations among crime rates, annual alcohol consumption, annual movie attendance, and the unemployment rate.

Given the small sample size (n = 12), we employed simple linear regression instead of more complex time-series models, which may be inappropriate due to limited degrees of freedom. We estimated two linear regression models for each crime type, with the crime rate as the dependent variable. The first model examined the impact of social distancing and economic factors, using annual movie attendance (in millions) as a proxy for social distancing and the unemployment rate as the economic indicator. The second model additionally included *per capita* alcohol consumption to examine how the association between social distancing and crime rates was attenuated when accounting for alcohol use.

To ensure the validity of the regression results, we assessed multicollinearity using variance inflation factors. We also conducted residual diagnostics, including normality tests, Q-Q plots, and residual plots, to verify that the underlying assumptions of the linear models were met. Additionally, we performed the Durbin-Watson (DW) test to check for potential autocorrelation, given the annual sequence of the data. Finally, to confirm that the findings were not solely attributed to the anomalous conditions of the COVID-19 pandemic, we conducted robustness checks by re-estimating the models after excluding the years 2020 and 2021.

We used SPSS, version 30.0 (IBM Corp., Armonk, New York, USA) and jamovi, version 2.3 (The jamovi project, Sydney, Australia) for all analyses. We considered a *P*-value of <0.05 statistically significant.

## RESULTS

**Figure 1:**
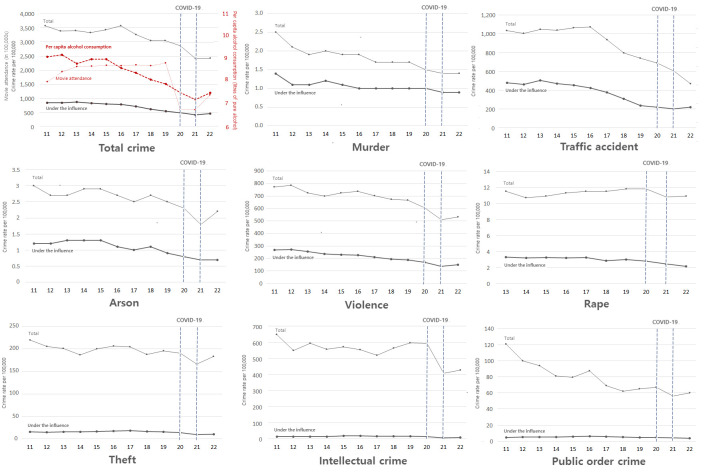
Annual crime rate and alcohol consumption *per capita* (2011−2022). **Panel A.** Total crime. **Panel B.** Murder. **Panel C.** Traffic accident. **Panel D.** Arson. **Panel E.** Violence. **Panel F.** Rape. **Panel G.** Theft. **Panel H.** Intellectual crime. **Panel I.** Public order offence.

Overall crime rates and offences committed under the influence of alcohol, which had been gradually declining since 2011, fell sharply during the COVID-19 pandemic (**Figure 1**, Panel A). This decline was observed across most crime categories, including traffic accidents, violence, murder, arson, and rape (**Figure 1**, Panels B–F). As of 2022, approximately 19% of all crimes were alcohol-related. This proportion was notably higher in murders (64%), traffic accidents (47%), arson cases (32%), violence (28%), and rape (20%). In contrast, lower proportions were observed in theft (6%), intellectual crimes (2%), and public order offences (6%) (**Figure 1**, Panels G–I).

Both *per capita* annual alcohol consumption and movie attendance declined sharply during the COVID-19 pandemic (2020–2021). Movie attendance showed a steeper drop, followed by a slight rebound in the second year. While overall crime rates, including alcohol-related crimes, also decreased during the pandemic, alcohol-related crimes more closely mirrored the trends in alcohol consumption and movie attendance than total crime rates.

According to the correlation analysis, while movie attendance and alcohol consumption were positively correlated with crime rates, alcohol consumption exhibited a stronger positive correlation (**Table 1**). Alcohol consumption showed a significant positive correlation with all crime rates, particularly with those committed under the influence of alcohol, most notably overall crime, murder, violence, and traffic accidents. Rape rates were significantly correlated with alcohol consumption only in alcohol-related cases. Movie attendance was positively associated with overall crime, violence, traffic accidents, and arson in both the total and alcohol-related crime categories. However, rape, theft, intellectual crimes, and public order offences were significantly correlated with movie attendance only when the offences were committed under the influence of alcohol.

**Table 1:** Correlations among alcohol consumption, social distancing, and crime rates

	Alcohol consumption		Movie attendance		Unemployment rate	
	**r**	***P*-value**	**r**	***P*-value**	**r**	***P*-value**
**Movie attendance**	0.692	0.013				
**Unemployment rate**	−0.295	0.351	−0.061	0.849		
**Total crime**						
Total	0.907	<0.001	0.723	0.008	0.010	0.976
UIA	0.977	<0.001	0.687	0.014	−0.297	0.348
**Murder**						
Total	0.884	<0.001	0.474	0.119	−0.223	0.484
UIA	0.907	<0.001	0.552	0.062	−0.235	0.461
**Murder (completed)**						
Total	0.823	0.001	0.456	0.136	−0.214	0.504
UIA	0.885	<0.001	0.575	0.050	−0.219	0.493
**Murder (attempted)**						
Total	0.896	<0.001	0.437	0.155	−0.211	0.510
UIA	0.903	<0.001	0.429	0.163	−0.298	0.347
**Rape**						
Total	-0.170	0.637	0.126	0.727	0.463	0.177
UIA	0.858	0.001	0.760	0.011	0.247	0.491
**Violence**						
Total	0.927	<0.001	0.735	0.006	−0.083	0.796
UIA	0.966	<0.001	0.643	0.024	−0.297	0.348
**Traffic accident**						
Total	0.924	<0.001	0.693	0.012	−0.027	0.932
UIA	0.965	<0.001	0.632	0.027	−0.347	0.268
**Arson**						
Total	0.909	<0.001	0.735	0.006	−0.136	0.673
UIA	0.935	<0.001	0.737	0.006	−0.168	0.600
**Theft**						
Total	0.747	0.005	0.535	0.073	−0.137	0.671
UIA	0.625	0.030	0.825	0.001	0.299	0.345
**Intellectual crime**						
Total	0.598	0.040	0.458	0.134	0.220	0.491
UIA	0.491	0.104	0.767	0.004	0.403	0.193
**Public order offence**						
Total	0.822	0.001	0.308	0.329	−0.363	0.246
UIA	0.678	0.015	0.780	0.003	0.241	0.451

UIA – under the influence of alcohol

A regression analysis was performed to assess the impact of social distancing on crime rates during the COVID-19 pandemic, using movie attendance and the unemployment rate as independent variables (**Table 2**). In both the general population and alcohol-related cases, overall crime, violence, traffic accidents, and arson were positively associated with movie attendance (used as a proxy for social interaction). For rape, theft, intellectual crimes, and public order offences, the positive association emerged only in alcohol-related cases.

**Table 2:** Regression analysis of social distancing and crime rates

	Total		Murder		Murder (completed)		Murder (attempted)		Rape		Violence		Traffic accident		Arson		Theft		Intellectual crime		Public order offence	
	***B* (SE)**	***P*-value**	***B* (SE)**	***P*-value**	***B* (SE)**	***P*-value**	***B* (SE)**	***P*-value**	***B* (SE)**	***P*-value**	***B* (SE)**	***P*-value**	***B* (SE)**	***P*-value**	***B* (SE)**	***P*-value**	***B* (SE)**	***P*-value**	***B* (SE)**	***P*-value**	***B* (SE)**	***P*-value**
**Total population**																						
Constant	2105.175 (1048.569)	0.076	2.080 (1.040)	0.076	1.271 (0.754)	0.126	0.813 (0.359)	0.050	9.112 (1.470)	<0.001	536.782 (223.854)	0.040	436.457 (573.045)	0.466	2.242 (0.846)	0.026	190.655 (44.068)	0.002	269.465 (225.287)	0.262	135.387 (65.109)	0.067
Movie attendance in millions	4.590 (1.454)	0.012	0.002 (0.001)	0.142	0.002 (0.001)	0.160	0.001 (0.000)	0.181	0.001 (0.002)	0.666	1.006 (0.310)	0.010	2.292 (0.795)	0.018	0.004 (0.001)	0.010	0.116 (0.061)	0.091	0.520 (0.312)	0.131	0.088 (0.090)	0.358
Unemployment rate	67.196 (282.255)	0.817	−0.191 (0.280)	0.513	−0.130 (0.203)	0.537	−0.061 (0.097)	0.545	0.560 (0.394)	0.198	−10.179 (60.257)	0.870	9.843 (154.253)	0.951	−0.093 (0.228)	0.694	−4.429 (11.862)	0.718	53.130 (60.643)	0.404	−20.445 (17.526)	0.273
R^2^	0.53		0.26		0.24		0.23		0.23		0.54		0.48		0.55		0.30		0.27		0.21	
**Under the influence**																						
Constant	841.378 (433.333)	0.084	0.762 (0.522)	0.178	0.526 (0.388)	0.208	0.261 (0.157)	0.130	1.030 (0.960)	0.319	259.436 (121.519)	0.062	556.891 (319.059)	0.115	0.901 (0.582)	0.156	−1.315 (4.437)	0.774	−11.318 (6.133)	0.098	1.692 (1.274)	0.217
Movie attendance in millions	1.777 (0.601)	0.016	0.001 (0.001)	0.077	0.001 (0.001)	0.063	0.000 (0.000)	0.185	0.004 (0.001)	0.010	0.439 (0.169)	0.028	1.144 (0.442)	0.029	0.003 (0.001)	0.009	0.035 (0.006)	<0.001	0.045 (0.009)	0.001	0.008 (0.002)	0.002
Unemployment rate	−131.301 (116.645)	0.289	−0.105 (0.140)	0.473	−0.072 (0.104)	0.506	−0.040 (0.042)	0.368	0.329 (0.257)	0.241	−35.129 (32.711)	0.311	−112.274 (85.885)	0.224	−0.087 (0.157)	0.591	2.843 (1.194)	0.041	4.930 (1.651)	0.015	0.537 (0.343)	0.152
R^2^	0.54		0.35		0.37		0.26		0.66		0.48		0.50		0.56		0.80		0.79		0.69	

*B* – unstandardized coefficient, SE – standard error

When *per capita* alcohol consumption was included in the model (**Table 3**), the previously observed associations between movie attendance and the rates of overall crime, rape, violence, traffic accidents, and arson were attenuated and replaced by strong positive associations with alcohol consumption. Moreover, the rates of both attempted and completed murder, which had not shown significant associations with movie attendance in the initial models, were significantly and positively associated with alcohol consumption.

**Table 3:** Regression analysis of social distancing, alcohol consumption, and crime rates

	Total		Murder		Murder (completed)		Murder (attempted)		Rape		Violence		Traffic accident		Arson		Theft		Intellectual crime		Public order offence	
	***B* (SE)**	***P*-value**	***B* (SE)**	***P*-value**	***B* (SE)**	***P*-value**	***B* (SE)**	***P*-value**	***B* (SE)**	***P*-value**	***B* (SE)**	***P*-value**	***B* (SE)**	***P*-value**	***B* (SE)**	***P*-value**	***B* (SE)**	***P*-value**	***B* (SE)**	***P*-value**	***B* (SE)**	***P*-value**
**Total population**																						
Constant	−2589.425 (919.550)	0.023	−2.415 (1.039)	0.049	−1.65 (0.964)	0.125	−0.818 (0.292)	0.023	12.214 (2.930)	0.006	−454.246 (205.795)	0.058	−2177.096 (458.393)	0.001	−1.245 (0.962)	0.232	55.805 (70.971)	0.454	−396.071 (370.355)	0.316	−144.972 (65.944)	0.059
Movie attendance in millions	0.655 (0.933)	0.502	−0.001 (0.001)	0.208	−0.001 (0.001)	0.412	−0.001 (0.000)	0.061	0.004 (0.003)	0.256	0.175 (0.209)	0.426	0.102 (0.465)	0.833	0.001 (0.001)	0.389	0.002 (0.072)	0.973	−0.038 (0.376)	0.922	−0.147 (0.067)	0.059
Unemployment rate	353.525 (136.890)	0.032	0.083 (0.155)	0.604	0.048 (0.143)	0.748	0.039 (0.043)	0.399	0.474 (0.388)	0.267	50.265 (30.636)	0.139	169.247 (68.240)	0.038	0.120 (0.143)	0.426	3.796 (10.565)	0.729	93.722 (55.133)	0.128	−3.346 (9.817)	0.742
Alcohol consumption	523.516 (87.716)	<0.001	0.501 (0.099)	0.001	0.326 (0.092)	0.008	0.182 (0.028)	<0.001	−0.408 (0.337)	0.271	110.514 (19.631)	<0.001	291.449 (43.726)	<0.001	0.389 (0.092)	0.003	15.038 (6.770)	0.057	74.217 (35.328)	0.069	31.264 (6.290)	0.001
R^2^	0.91		0.82		0.71		0.88		0.39		0.91		0.92		0.86		0.57		0.53		0.81	
**Under the influence**																						
Constant	−1197.525 (277.937)	0.003	−1.475 (0.536)	0.025	−1.045 (0.457)	0.052	−0.455 (0.124)	0.006	−2.67 (1.252)	0.077	−303.763 (87.954)	0.009	−921.379 (231.437)	0.004	−1.626 (0.572)	0.022	−11.839 (7.977)	0.176	−18.750 (12.177)	0.162	−1.874 (2.152)	0.409
Movie attendance in millions	0.069 (0.282)	0.814	0.000 (0.001)	0.454	0.000 (0.000)	0.711	0.000 (0.000)	0.051	0.001 (0.001)	0.660	−0.033 (0.089)	0.722	−0.095 (0.235)	0.696	0.001 (0.001)	0.384	0.026 (0.008)	0.011	0.038 (0.012)	0.015	0.005 (0.002)	0.068
Unemployment rate	−6.946 (41.376)	0.871	0.031 (0.080)	0.706	0.023 (0.068)	0.739	0.004 (0.018)	0.845	0.432 (0.166)	0.040	−0.779 (13.093)	0.954	−22.113 (34.453)	0.539	0.067 (0.085)	0.456	3.485 (1.187)	0.019	5.383 (1.813)	0.018	0.754 (0.320)	0.046
Alcohol consumption	227.367 (26.512)	<0.001	0.249 (0.051)	0.001	0.175 (0.044)	0.004	0.080 (0.012)	<0.001	0.486 (0.144)	0.015	62.805 (8.390)	<0.001	164.848 (22.077)	<0.001	0.282 (0.055)	0.001	1.174 (0.761)	0.162	0.829 (1.162)	0.496	0.398 (0.205)	0.089
R^2^	0.96		0.84		0.79		0.89		0.88		0.94		0.94		0.90		0.85		0.81		0.79	

*B* – unstandardized coefficient, SE – standard error

We assessed the statistical validity of the regression analysis results with several diagnostic tests. Variance inflation factor values ranged from 1.0 to 2.2, indicating no significant multicollinearity. Residual diagnostics, including normality tests, Q-Q plots, and residual plots, confirmed that the models satisfied the fundamental assumptions of linear regression (Figure S1 in the **Online Supplementary Document**). The DW test confirmed the absence of significant autocorrelation in the primary models for total crime rate (DW = 2.79; *P* = 0.572) and total crime rate committed under the influence of alcohol (DW = 2.61; *P* = 0.824). Finally, we conducted robustness checks by re-estimating all models after excluding the pandemic years (2020–2021). The results remained consistent in terms of both direction and statistical significance (Tables S2 and S3 in the **Online Supplementary Document**).

## DISCUSSION

We examined changes in crime rates and alcohol consumption during the COVID-19 pandemic, with a focus on the impact of social distancing measures. Both alcohol use and social interaction declined during this period. Trends in alcohol consumption were closely aligned with the rates of overall crime, murder, traffic accidents, arson, and violence. While social distancing was significantly associated with crime rates in models that excluded alcohol consumption, this association largely disappeared when alcohol use was included. In contrast, alcohol consumption remained a significant predictor of crime rates across most categories. These results indicate that the relationship between social distancing and certain crimes may be functionally linked through alcohol consumption patterns.

We highlighted three key findings. First, the decline in alcohol consumption observed in many countries during the COVID-19 pandemic can be partially attributed to social distancing. The pandemic triggered social and environmental changes that influenced alcohol use in complex ways, including a negative impact on mental health and a reduction in social gatherings [36]. Although global trends indicated a general decrease in alcohol consumption [24], local patterns varied, with some countries reporting increases [37–40] and others reporting decreases [41–43]. Our findings suggest that, in specific contexts, the reduction in alcohol consumption during the pandemic might have been substantially driven by social distancing. Moreover, the extent of this effect may depend on the cultural significance of alcohol in social settings, as demonstrated in previous studies [44,45].

Second, although the association between alcohol and crime is well established at both the individual and population levels, we suggest that the relationship depends on the patterns of consumption as well as the quantity of alcohol consumed. The finding that the initial association between social distancing and crime rates was significantly attenuated once alcohol consumption was accounted for implies that crime rates can be substantially influenced by the social regulation of drinking. While social distancing involved compulsory public health measures, the observed variation in alcohol consumption suggests that culturally informed alcohol policies may hold significance, particularly in societies where the social context of drinking is deeply rooted.

By positioning South Korea as a culture with high levels of social drinking, our findings offer preliminary insights for global policymakers regarding alcohol policies. In societies where alcohol consumption is deeply intertwined with social interaction, alcohol policies need to go beyond general availability or price controls by targeting the ‘socially facilitated’ nature of drinking. Our results also suggest that policies regulating social drinking environments, such as restricting on-premises consumption hours or redesigning high-density social drinking districts, could be considered alongside traditional law enforcement in reducing alcohol-related crimes in similar cultural contexts worldwide.

Third, we identified specific types of crime strongly associated with alcohol use. While theft and intellectual crimes showed no significant association with alcohol consumption, violent offences, such as murder, rape, traffic accidents, and arson, were significantly linked to alcohol intoxication. These findings align with previous research indicating a stronger association between alcohol intoxication and violent crimes compared to non-violent ones [3,46]. Both qualitative and quantitative studies have established that alcohol significantly increases aggression [47], and the found association between alcohol and violent crimes is consistent with alcohol’s potential role in aggressive behaviour.

We highlight variations in the context of alcohol consumption depending on the type of crime. Except for murder, which was significantly associated with alcohol consumption but not with social distancing, the rates of violence, rape, traffic accidents, and arson were initially associated with social distancing. However, when alcohol consumption was included in the regression model, these associations were instead statistically accounted for by alcohol consumption. Our findings suggest that the association between alcohol and crime is closely tied to socially contextualised drinking. While alcohol consumption may play a role in murder, its lack of association with social drinking implies that such crimes are more likely linked to heavier, solitary drinking rather than socially motivated alcohol use. This is consistent with previous findings that assault is more commonly associated with social drinking, whereas murder is more frequently linked to private drinking [48]. The significant association of other serious crimes, such as violence, rape, accidents, and arson, with socially facilitated alcohol consumption substantiates the importance of targeting social alcohol use in crime prevention strategies.

This study has several limitations that constrain a deeper analysis of the relationship between alcohol consumption and crime, restricting a ‘dose-response’ analysis. First, the data lacked detailed information on the level of intoxication, indicating only whether the suspect was intoxicated, without quantifying the degree of intoxication. However, given that alcohol consumption itself may have greater relevance than the exact quantity consumed and that intoxication is often determined based on circumstantial evidence in the crime statistics of many countries [49–51], our classification serves as a meaningful indicator of alcohol involvement.

Second, while the data from the National Tax Service comprehensively capture alcohol consumption in Korea, including both domestic and imported alcoholic beverages, it does not cover duty-free alcohol, the use of which could have been affected during the pandemic. Moreover, a decline in overall consumption figures during the pandemic may not fully account for the potential displacement of drinking from public venues to private settings. However, given the comprehensive coverage of the National Tax Service data, the very low rate of unrecorded alcohol consumption (reportedly <0.5%) [52], and the strict regulations on duty-free allowances in Korea, our data can be considered a reliable and faithful measure of alcohol consumed in the country.

Third, although movie attendance was highly sensitive to social distancing during the pandemic, it may have been further affected by cinema closures, industry changes, and substitution with streaming services. Although these factors are interlinked to social distancing as secondary consequences of the reduced movie attendance, they may also have an independent and additive impact on movie attendance. However, given its high correlation and explanatory power compared to other available proxy measures, movie attendance remains a reliable proxy for discretionary social interaction.

Lastly, the high R^2^ values in our models may have partially resulted from the short study period of 12 years, making the models prone to overfitting. The ecological design and the small number of annual observations limit causal or mediational inferences; however, robustness checks (excluding 2020–2021) indicate that the associations are stable and not driven by pandemic-related structural breaks. In addition, variance inflation factor values and residual diagnostics indicated no significant multicollinearity or violations of regression assumptions. This suggests that the observed role of alcohol consumption represents a substantive association rather than a statistical artefact. Therefore, our findings provide meaningful insights into the relationship between social distancing, alcohol consumption, and crime.

## CONCLUSIONS

The concurrent declines in crime and alcohol consumption, along with the attenuating effect of alcohol on the relationship between social distancing and crime, suggest that changes in social drinking opportunities may be associated with shifts in specific types of violent crime. Although the unique pandemic conditions – including simultaneous changes in mobility, policing, and reporting – preclude definitive causal conclusions, these findings provide a preliminary basis for considering how shaping drinking contexts may contribute to crime prevention. Future research using more rigorous designs is needed to isolate these mechanisms from other pandemic-related factors. Nevertheless, our findings highlight the potential relevance of policies that shape drinking contexts, such as regulating alcohol availability in restaurants and pubs and addressing workplace drinking norms. Further exploration of alcohol-related media exposure and advertising regulations may also be warranted within broader public health strategies. These approaches may be particularly relevant in cultural contexts where social drinking is prevalent.

## Additional material


Online Supplementary Document
